# Conference Reports: CONFERENCE ON FIFTY YEARS WITH NUCLEAR FISSION Washington, DC and Gaithersburg, MD April 25–28, 1989

**DOI:** 10.6028/jres.095.022

**Published:** 1990

**Authors:** Oren A. Wasson

**Affiliations:** Ionizing Radiation Division, Center for Radiation Research, National Institute of Standards and Technology, Gaithersburg, MD 20899

The National Institute of Standards and Technology (NIST) and the American Nuclear Society jointly sponsored this unique conference to celebrate the fiftieth anniversary of the discovery of nuclear fission. An international audience of over 400 scientists and engineers joined the distinguished early pioneers to reminisce about the past and to study recent developments of this important technological discovery. The conference highlighted the early pioneers of the nuclear community by dedicating a full day of plenary presentations at the National Academy of Sciences in Washington, DC and later at the conference banquet. More recent developments in fission science and technology in addition to historical reflections were topics for two full days of sessions at NIST in Gaithersburg, MD. The meeting was extremely fortunate in having Professors John A. Wheeler and Edoardo Amaldi serve as Honorary Co-Chairmen and in having Professors Glenn T. Seaborg and Emilio Segre serve as General Co-Chairmen. The conference attendees were saddened by the death of Professor Segrè on April 22, 1989, just three days before the start of the conference.

## 1. Opening the Conference

The conference was opened by welcoming remarks by Raymond G. Kammer, acting director of NIST and Gail de Planque, president of the American Nuclear Society. In his remarks, Kammer noted the relation of the National Bureau of Standards to the development of fission energy. Nuclear fission was discovered in Germany in late 1938 by Hahn and Strassmann. The news was brought to the United States in January of 1939 by Neils Bohr. Within days the discovery was announced in Washington, DC at a conference on theoretical physics at George Washington University. The results were soon repeated at the Department of Terrestrial Magnetism and at other laboratories throughout the nation.

The announcement at the Washington conference made the front page of the January 26, 1939 edition of the Washington Evening Star newspaper under the headline “Power of New Atomic Blast Greatest Achieved on Earth” with the subtitle “Physicists Here Hail Discovery Greatest Since Radium.” After an extensive discussion of the physics of the process, the author stated that “the practical uses of the discovery remain vague. It may place hitherto undreamed-of resources in the hands of physicists for experimental purposes and help clear up some of the outstanding mysteries of creation.” And finally the article concluded with the statement “As a practical power source, the new finding has at present no significance.”

It is interesting that the director of the National Bureau of Standards, Lyman Briggs, was appointed by President Roosevelt to chair the first federal government committee to study the possible uses of this new development in nuclear science. The first meeting of the Advisory Committee on Uranium met at the Department of Commerce on October 21, 1939. The meeting included Professor Teller who was the author of a contribution to this conference. The report, dated November 1, 1939, stated that a chain reaction was a possibility. It speculated on the possibility of a new explosive for the military as well as a new source of energy for submarines for the navy. The report recommended that 4 tons of pure graphite be obtained at once for research and that later 50 tons of uranium ore be acquired. Three months later $6000 was made available to purchase a small quantity of graphite for experiments. Thus began the government support for the start of the immense enterprise of nuclear energy.

## 2. Conference Topics

The wide range of topics covered by this meeting included plenary sessions entitled:

Prelude to the First Chain Reaction—1932 to 1942
Early Fission Research—Nuclear Structure and Spontaneous Fission
50 Years of Fission, Science, and Technology
Nuclear Reactors, Secure Energy for the Future
Nuclear Fission—A Prospective,
invited sessions entitled:
  Reactors
  Fission Science
  Safeguards and Space Applications
  Fission Data
  Nuclear Fission—Its Various Aspects
  Medical and Industrial Applications of By-Products,

and contributed (both oral and poster) sessions on:

Theory and Experiments in Support of Theory
Reactors and Safeguards
General Research, Instrumentation, and By-Products
Fission Data, Astrophysics, and Space Applications.

## 3. Summary of Presentations on the Early Work on Fission

The titles and authors, along with a summary of several of the papers presented on the early work on the discovery of fission and applications of nuclear energy are given in this section.

1. “The Prelude to Fission, Italy,” Edoardo Amaldi (University of Rome-Italy)
  After the discovery by Irene Curie and Frederic Joliot of artificial radioactivity induced by alpha particles, Fermi thought that similar effects could be observed also by using neutrons. In 1934 Fermi and co-workers (Amaldi, D’Agostino, Rasetti, Segrè) irradiated 60 elements with neutrons, found 44 new radioactive bodies, and in 16 cases, identified the chemical nature of the product of the reaction. They demonstrated that two artificial radioactive bodies produced in U, one of 13 min (later 15 min) half-life, the other about 100 min half-life, were not due to elements with atomic number between 82 and 92. Fermi et al. suggested that these bodies could be radioisotopes of transuranic elements of Z = 93 and 94 produced by neutron capture in ^238^U followed by two successive beta decays.
2. “The Prelude to Fission, France,” P. Savić (Serbian Academy of Sciences and Arts-Yugoslavia)
  A personal account of the events leading to the 1938 discovery, by Irene Joliot Curie and the author, of the unidentified element R_3.5h_ under neutron bombardment of uranium was presented, as well as the experimental methods proving that R_3.5h_ had chemical properties similar to lanthanum. This in turn led to Hahn and Strassmann’s discovery of fission in 1939.
3. “How Fission Was Discovered,” Siegfried Fluegge (University of Freiberg-Federal Republic of Germany)
  After the great survey of neutron-induced radioactivity by Fermi and coworkers, the laboratories in Paris and Berlin-Dahlen tried to disentangle the complex results found in uranium. At that time neutron sources were small, activities low, and equipment very simple. Chemistry beyond uranium still was unknown. Hahn and Meitner believed they had observed three transuranic isomeric chains, a doubtful result even then. Early in 1938, Curie and Savić in Paris found an activity interpreted to be actinium, and Hahn and Meitner another to be radium. Both interpretations seemed impossible from energy considerations. Hahn and Strassmann, therefore, continued this work and succeeded in separating the new activity from radium. There remained no doubt that a barium isotope had been produced, the uranium nucleus splitting in the yet-unknown process we now call fission.
4. “The Early French Program,” Bertrand Goldschmidt (Commission L’ Energie Atomic, Paris-France)
  The work of Joliot’s team from 1939 to mid-1940, a physical proof of fission, the reaction to Szilard’s proposal of secrecy, the detection of the secondary neutrons and their quantitative measurements, the taking-out of secret patents, the tentative agreement with Union Miniere du Haut Katanga and the procurement of uranium, the search for a moderator, the purchase of the worldwide stock of heavy water from Norway and its transfer to England in June 1940 were highlighted.
5. “Early Work in Copenhagen and in England,” Rudolf Peierls (Nuclear Physics Laboratory-England)
  Starting from the insight by O. R. Frisch and Lise Meitner, the talk reviewed Frisch’s first observation of fission fragments, which caused him to propose the term “fission.” This was followed by an account of the argument which led Niels Bohr to the realization that the slow-neutron fission was due to ^235^U, and his conclusion that no explosive reaction was possible in natural uranium. The Frisch-Peierls memorandum, which suggested that the critical mass of ^235^U was much less than suspected and that the assembly of a supercritical amount of ^235^U would lead to an explosive reaction with a high yield, was presented.
6. “Experiments at Columbia and The University of Chicago which Led to the First Chain Reaction,” Walter Zinn (GNEC-retired)
  The author offered his recollections of the experimental efforts beginning in 1939 which culminated in the Chain Reaction in the squash court on December 2, 1942. Recalled were Columbia University experiments which did much to establish the feasibility of the chain reaction in natural uranium and which stimulated the creation of the Manhattan District. The Columbia group moved to the University of Chicago, where, in early summer of 1942, construction and analysis of a number of subcritical reactors (piles) gave assurance with a high probability that only a reasonable amount of uranium and moderator would be required.
7. “Fission in 1939: The Puzzle and the Promise,” John Archibald Wheeler (Princeton University and University of Texas at Austin)
  How come fission? Above all, how does it come about that thermal neutrons and neutrons of energies above a couple of MeV are good at inducing fission in uranium, but not neutrons of intermediate energy? Bohr’s 1935–1937 compound-nucleus concept of nuclear reactions proved itself the key to this 1939 puzzle. To turn the key in the lock it was necessary to establish and exploit the concept of “fission barrier,” an idea contested initially by more than one colleague. To add a slow neutron to the even-neutron nucleus ^238^U does not produce enough excitation to surmount the barrier, but addition to the odd neutron ^235^U does. The barrier theory proposed this, and his colleagues confirmed it a year later, which signalled a new world of activity. The same argument said that ^239^Pu must be fissile, a circumstance of which Louis A. Turner was the first to point out the fantastic alchemical promise, to be released in Du Pont’s 1944–1945 deliveries of plutonium in many-kilogram amounts to Los Alamos.
8. “Soviet Research into Nuclear Fission Before 1941,” G. N. Flerov (Joint Institute for Nuclear Research-USSR)
  Some material pertaining to research aimed at clarifying the possibility of inducing the chain reaction was presented. Both theoretical and experimental studies were carried out to test different methods of producing the reaction and to analyze its kinetics. The high-sensitivity technique designed for detecting uranium fission permitted the discovery of the spontaneous fission of uranium and the later searches for the spontaneous fission of thorium. Subsequently this made it possible to assess the role of spontaneously fissioning nuclides in the occurrence of the uncontrolled chain reaction.
9. “The Early Japanese Program,” Paul Kuroda (University of Nevada, Las Vegas)
  Aston and Bohr visited and lectured in Japan in 1936 and 1937, respectively. Their visits to Japan shortly before WWII had a profound effect on Japanese scientists. The early Japanese program was led by Yoshio Nishina at the Institute of Physical and Chemical Research and Kenjiro Kimura at the Tokyo Imperial University during the period between 1938 and 1942. This work resulted in the discoveries of symmetric fission and a member of the “missing” radioactive family 4n + 1, ^237^U. In addition to the research carried out by the Nishina group, a physicist at the Kyoto Imperial University named Tokutaro Hagiwara proposed, as early as in May 1941, an extremely interesting idea of using a nuclear chain reaction to initiate a thermonuclear reaction.
10. “Reminiscences of Berlin and Chalk River,” Leslie G. Cook (ERE-retired)
  Personal reminiscences from the Kaiser Wilhelm Institute in Berlin, 1937–1938 were given. The course of experimental events and the continuing search for interpretations that would stand up, the guiding influence of the work of Irene Curie and Pavle Savìc on the experimental work of Hahn and Strassmann, the influence and effects of Nazi political pressures within the Institute, the shadows of war, and how uranium fission finally got itself discovered were explained. The talk continued with personal reminiscences from the Nuclear project in Canada.
11. “Spontaneous Fission of the Heaviest Elements,” Darleane C. Hoffmann (Lawrence Berkeley Laboratory, California)
  Although spontaneous fission was discovered in ^238^U by Petrzhak and Flerov in 1940, detailed studies of the process were first made possible in the 1960s with the availability of milligram quantities of ^252^Cf. However, until 1971 it was believed that the main features of the mass and kinetic-energy distributions were essentially the same as those for thermal neutron-induced fission and that all low-energy fission proceeded via asymmetric mass division with total kinetic energies which could be derived by linear extrapolation from those of lighter elements. Measurements for the heavier elements have shown symmetric mass distributions with both high and low total kinetic energies. Recent results for spontaneous fission properties of the heaviest elements were reviewed and compared with theory.
12. “Nuclear Fission and the Transuranium Elements,” Glenn T. Seaborg (Lawrence Berkeley Laboratory, California)
  Neutron irradiation of uranium in the 1930s led to the reported discovery of such transuranium elements as ekarhenium, etc. After a few years of investigation these were correctly identified as fission products. Not until it was recognized that the first four of the real transuranium elements should be part of a 14-member “actinide series” could elements 95 and 96 (americium and curium) be chemically identified following their nuclear synthesis. This new view was the key to the synthesis and identification of the next seven transuranium elements, resulting in the completion of the “actinide series” at element 103 in 1961. The “transactinide elements” could, according to the actinide concept, also be correctly placed in the Periodic Table and the chemical properties of the first transactinide elements, elements 104 and 105 (rutherfordium and hahnium), confirm this point of view. Transuranium elements through atomic number 109 are now known. There are possibilities for the discovery of more transactinide elements.
13. “Fission Technology: Retrospect and Prospect,” Alvin M. Weinberg (Institute for Energy Analysis)
  Forty-five years have passed since the “New Piles Committee” at Chicago first deliberated on the future of nuclear power. The light water reactor, first conceived at that time, now dominates the world’s fleet of 500-odd civilian power reactors. Though nuclear power now accounts for almost 8% of the world’s primary energy, its future hangs in doubt in many countries because of the public’s apprehensions over reactor safety and waste disposal. Yet the greenhouse threat adds great urgency to widespread deployment of nuclear power. Nuclear technologists are therefore challenged to develop nuclear systems that are fully acceptable to a skeptical public.
14. “The Future of Nuclear Reactors,” Edward Teller (Hoover Institute and Lawrence Liver-more National Laboratory)
  Measures to assure the safety of nuclear reactors in the United States began more than 40 years ago, and they have produced an unrivaled record of safe energy generation. While many nations are rapidly converting to nuclear-generated electricity, usage is declining in the United States, where misplaced public concerns and their political consequences have produced counterproductive and economically harmful regulations. Three innovative reactor designs—the modular high-temperature gas reactor, the PIUS, and the Geyser—offer smaller size and increased simplicity. Those factors, possibly combined with underground location, would provide unmistakable and convincing evidence of safety and could lead to replacing current requirements with practical, safety-enhancing regulations.
15. “Radioiodine: The Atomic Cocktail,” Rosalyn S. Yalow (Veterans Administration Medical Center)
  The use of artificial radionuclides in medicine has continued to increase in importance resulting in the growth of a new medical specialty, Nuclear Medicine. The availability of very low cost radionuclides from Oak Ridge, beginning in 1946, initiated a revolution that led to widespread use of ^131^I in the understanding and management of thyroid disease and to extensive use of ^131^I-labeled dyes, fats, drugs, proteins, and other substances in diverse areas of medicine. While the role of the “atomic cocktail” in cancer therapy has diminished greatly, *in vivo* and *in vitro* radionuclide procedures in medical diagnosis are employed in over one-third of hospital admissions.

## 7. Publication

The proceedings of the conference were published in a two-volume set under the title:

FIFTY YEARS WITH NUCLEAR FISSION
Edited by James W. Behrens and Allan
D. Carlson
Published by the American Nuclear Society, Inc.
La Grange Park, IL 50525

Also available from the publisher are video tapes of the presentations of the invited speakers as well as the talks of the banquet speakers.

